# Anticipatory gaze in a reaching-and-grasping task when target movement direction is uncertain: evidence of statistical learning

**DOI:** 10.3389/fpsyg.2026.1625415

**Published:** 2026-02-16

**Authors:** Youssef G. Ekladuce, Ryan W. Langridge, Jonathan J. Marotta

**Affiliations:** Perception and Action Lab, Department of Psychology, University of Manitoba, Winnipeg, MB, Canada

**Keywords:** anticipatory gaze, directionality bias, reaching-to-grasp, uncertainty, visuomotor control

## Abstract

Successful goal-directed actions in dynamic environments depend on the brain’s ability to implicitly learn probabilistic regularities, enabling anticipatory behaviors and reducing cognitive demands. Humans can utilize visual and non-visual aspects about the target to predict its direction. We investigated whether individuals could implicitly exploit a target’s directional movement history during a grasping task to predict its future direction, under uncertain conditions, lacking any clues about the target’s eventual movement. In each trial, a target appeared in the middle of the screen, remained stationary for 2.50 s, then translated horizontally toward the left or the right. Participants first completed a non-biased block of trials, before completing either two rightward or leftward biased blocks. Upon exposure to the biased blocks, participants’ average gaze shifted toward the direction of the eventual movement of the target prior to its movement, suggesting an adaptation in gaze strategy as the experiment progressed. Later trials showed a greater distance from the target’s horizontal midline compared to earlier trials. Furthermore, anticipatory behavior facilitated more efficient reactive gaze adjustments once the target began moving, enabling participants to align their gaze more closely with the target and thus reducing cognitive load. This study highlights the visuomotor system’s ability to implicitly use probabilistic patterns, enhancing anticipatory and reactive gaze strategies that improve the execution of goal-directed actions.

## 1 Introduction

Goal-directed behaviors, such as grasping a moving object, require the brain to process visual information to estimate the object’s position and velocity at the time of contact (Carnahan et al., 1993; Hesse et al., 2016; Paninski et al., 2004; Todd, 1981). For instance, when attempting to catch a ball, a soccer goalie must anticipate the direction that the ball will be kicked and initiate movements accordingly. However, through extensive practice, these anticipatory actions become increasingly automatic, allowing the goalie to exploit probabilistic estimations about the ball’s trajectory, thereby optimizing their predictive accuracy (Malla and López-Moliner, 2015; Mennie et al., 2007; Spering et al., 2011). Since our visual environment contains many statistical regularities (Turk-Browne, 2012), the brain’s capacity to implicitly learn these regularities enables the operation of actions, reducing cognitive load and enhancing the likelihood of successful goal-directed behavior, particularly in dynamic and uncertain situations (Masters and Maxwell, 2008).

The two-visual-stream hypothesis proposes that visual processing involves two functionally independent neural pathways, a dorsal visual stream and a ventral visual stream (Goodale et al., 1991; Goodale and Milner, 1992; Goodale, 2011, 2013). The dorsal visual stream projects to the posterior parietal cortex and processes visual information serving the representations needed for visually guided actions and is largely unconscious. In contrast, the ventral stream, projecting to the inferior temporal cortex, processes information consciously, relying on memory to support stable perceptual representations of the world around us (Goodale et al., 1991; Goodale and Milner, 1992; Goodale, 2011; Jakobson et al., 1991; Milner and Goodale, 2006; Miyashita, 1993). Despite their functional independence, research suggests that various types of goal-directed behavior require the communication of the dorsal and ventral visual streams. These behaviors include tool use (Brandi et al., 2014; Budisavljevic et al., 2018; Dressing et al., 2018), delayed grasping (Cohen et al., 2009; Singhal et al., 2013), and engaging with two-dimensional objects (Freud et al., 2018). These findings are significant as they suggest that in certain contexts, the non-conscious dorsal stream can access ventrally mediated conscious memory systems, gaining information not typically accessible to the dorsal stream alone, but that may aid visually guided actions, such as the ability to utilize an object’s history of movement to predict its future movement (Keizer et al., 2008; Monaco et al., 2019; Perry and Fallah, 2014).

Anticipating the direction of a moving object involves two steps: first, one must be able to identify the trajectory of the object (Krekelberg and Lappe, 1999; Montagne et al., 1999; Müller and Abernethy, 2006; Müller et al., 2006). Second, one must be able to look ahead into that path to maximize the chances of successfully catching the object (Bulloch et al., 2015; Spering et al., 2011; Thulasiram et al., 2020). Müller et al. (2006) examined how elite cricket batsmen anticipate a bowler’s delivery intentions in their sport. Their findings suggest that skilled players rely heavily on early visual cues, such as the bowler’s kinematic cues to predict the ball’s trajectory. Thus, this ability to anticipate based on early visual information is critical for intercepting a moving object in dynamic environments (Müller et al., 2006).

Once the trajectory of a moving object is anticipated, one could fixate ahead in the path of the object prior to grasping it. Bulloch et al. (2015) conducted a study to examine the effects of manipulating the direction of movement of two-dimensional targets on gaze behavior. To do so, participants were asked to reach and grasp a two-dimensional target that appeared on the leftward or rightward edge of the screen and translated horizontally. Results revealed that participants fixated about 7 cm ahead of the leading edge of the target prior to the onset of its movement (Bulloch et al., 2015). The researchers attributed these results to participants’ attempts to eliminate the “cognitive effort” that must be exerted to catch up to and continue following a target that has already begun moving (Bulloch et al., 2015). Similar results were observed in a study that utilized vertically moving two-dimensional targets (Thulasiram et al., 2020). Therefore, anticipating the direction and trajectory of a moving object appears to benefit visual pursuit of that object, and is crucially dependent on the ability to interpret and react to early visual information.

Anticipating the trajectory of a moving object could be achieved through the processing of more than just the low-level visual cues of the object’s movement. For instance, Diaz et al. (2013) found that when individuals are asked to hit a bouncing ball with a racquet, they perform anticipatory saccades in the future trajectory of the bounced ball prior to its bounce. This finding persisted even when the speed and elasticity of the ball were changed before it bounced, suggesting that participants could utilize information beyond what was immediately available to them to guide elements of the goal-directed behavior (Diaz et al., 2013). Similar anticipatory behavior has been observed in more naturalistic contexts, such as squash, where participants look ahead in the post-bounce trajectory of the ball before it contacts the wall (Hayhoe et al., 2012). In addition, Aglioti et al. (2008) demonstrated that elite basketball players can predict the fate of free-throw shots earlier and more accurately than visually experienced non-athletes by relying on subtle kinematic cues from the shooter’s body before the ball leaves the hand. Their motor system shows time-specific corticospinal modulation when observing erroneous shots, suggesting that extensive motor experience refines anticipatory mechanisms that support predicting the trajectory of the ball (Aglioti et al., 2008). Thus, it is evident that the visuomotor system relies on extensive, higher-level information, such as angle of incidence, body configuration of the actor, and future direction of movement, to anticipate the trajectory of a moving target. However, the extent to which the visuomotor system can navigate uncertain environments, where the only available information about the target is its statistical pattern of previous movement, is not yet known. In other words, can the visuomotor system utilize environmental statistics to produce anticipatory behavior when guiding actions?

There is a growing body of evidence that suggests that statistically regular events in our environment lead to the production of anticipatory behavior in perceptual contexts. Notaro et al. (2019) explored the impact of input statistics on anticipatory behavior using a statistical learning paradigm. By manipulating the probability of target location to be either 70% on the same side as the previous trial or 30%, they found that participants’ anticipatory saccades were biased toward the same side in the high-probability condition and toward the opposite side in the low-probability condition. These results indicate that participants adjusted their anticipatory gaze based on the pattern-based regularities provided, demonstrating that predictive oculomotor behavior can be shaped by learned statistical regularities and expressed prior to stimulus onset, independent of explicit directional cues. Recent findings further show that implicitly learned event-context associations can bias perceptual predictions. For example, Betti et al. (2022) used a probabilistic learning design in which contextual cues, such as color, were paired with actions or shapes at different co-occurrence probabilities, creating high-expectancy *(stronger learned association)* and low-expectancy *(weaker learned association)* contexts. Results showed that the learned contextual priors biased the participants’ predictions when asked to disambiguate events, particularly when visual information was limited and the contextual cue was more strongly predictive (Betti et al., 2022).

Additional studies have shown that probabilistic regularities also shape anticipatory eye movements during continuous visual tracking. For example, Damasse et al. (2018) investigated the influence of probabilistic information on anticipatory smooth pursuit eye movements by asking participants to track a moving target that appeared in the middle of the screen and translated horizontally toward the right or the left side of the screen. The frequency of the target’s movement to the right or left was manipulated, and participants exhibited a marked increase in anticipatory smooth eye velocities, reflecting a systematic adaptation to the direction bias (Damasse et al., 2018). Additional studies have also demonstrated that gaze-directed adaptations consistently emerge from implicit learning of directional frequency (e.g., Montagnini et al., 2010; Santos and Kowler, 2017; for review, please see Fiehler et al., 2019), suggesting the visual system is sensitive to probabilistic information regarding stimulus movement. Importantly, however, these studies have demonstrated humans’ sensitivity to directionality bias and the resulting production of anticipatory gaze behavior, in perceptual contexts that did not require goal-directed action, such as grasping the moving stimulus.

### 1.1 Objectives and hypotheses

Often, we pursue the movement of a target stimulus to intercept it, such as the goalie catching a soccer ball. Yet, it remains unknown whether the previously observed ability to exploit a target’s directionality bias to produce oculomotor anticipatory behavior is demonstrated when individuals are asked to reach to and grasp the moving target, in addition to simply tracking its eventual movement. In other words, can the visuomotor system effectively leverage implicit probabilistic information acquired from previous experiences to facilitate anticipatory behavior during the planning and execution of goal-directed actions?

The purpose of the present study was to investigate the visuomotor system’s ability to utilize the directionality bias of a moving target based on its movement history, to produce anticipatory gaze behavior when performing a reaching and grasping task. Participants were required to reach and grasp a horizontally moving target. In each trial, the target initially appeared at the center of the screen and then translated horizontally toward the right or the left. The horizontal movement of the target was manipulated so that in most trials, the target “preferentially favored” one direction, establishing a directionality bias. Participants were randomly assigned to one of two groups: a Rightward Bias group, in which the target more often moved to the right, and a Leftward Bias group, in which it more often moved to the left. Using biases in both directions allowed us to attribute any anticipatory gaze shifts to the experimental manipulation rather than to inherent asymmetries in spatial attention. This was important because leftward anticipatory gaze alone could be confounded with pseudoneglect, the tendency for neurotypical adults to allocate attention disproportionately toward the left visual field due to right-hemisphere lateralization of the parieto-frontal network (Thiebaut de Schotten et al., 2011). Finally, a between-subjects design was necessary to prevent participants from learning or carrying over the directionality bias across conditions.

Considering the literature demonstrating anticipatory gaze behavior during the pursuit of a moving target, and the potential for the memory of environmental probabilities stored within the ventral stream to be communicated to the dorsal stream’s guidance of grasping behavior, it was predicted that the visuomotor system would be sensitive to the directionality bias of a moving target, leading to production of anticipatory gaze behavior in the direction of the bias. More specifically, we hypothesized that in the absence of a directionality-bias (target moved an equal number of times toward the right and the left), participants would not demonstrate anticipatory gaze behavior but maintain an average gaze toward the horizontal midline of the stationary target (Desanghere and Marotta, 2011) due to the uncertain direction of its eventual movement. In contrast, in the presence of a directionality bias, we hypothesized that participants’ gaze behavior would shift toward the anticipated leading edge of the target while stationary. For instance, participants in the right-directionality-bias (Rightward Bias) group would direct their gaze toward the right edge of the target prior to the onset of its movement, with the opposite pattern would be observed in the left-directionality-bias (Leftward Bias) group. Furthermore, we hypothesized that as participants complete more directionality-biased trials, anticipatory behavior would increase in magnitude, and participants’ gaze would shift farther away from the midline of the target, toward the anticipated leading edge, thus suggesting that learning of the probabilistic patterns had taken place.

In addition, we hypothesized that anticipatory gaze behavior would systematically shape subsequent oculomotor reactionary behavior once the target began moving. Specifically, we predicted that greater anticipatory gaze in the direction of the eventual target movement would reduce the spatial and temporal demands on initial reactive eye movements, such that the amplitude and duration of the first catch-up saccade following target movement onset would decrease as anticipatory behavior increased, and that the first fixation following this saccade would fall closer to, or slightly ahead of, the target’s midline, reflecting more efficient tracking. Finally, we hypothesized that these changes in oculomotor behavior would be accompanied by adaptations in movement kinematics across the two biased trial blocks: as participants learned and exploited the directionality bias, reaching movements were expected to become more efficient, characterized by subtle adjustments in peak reaching velocity, reach duration, and reach latency that would reflect a reduced need for corrective movements once the target was in motion.

## 2 Materials and methods

### 2.1 Participants

Fifty individuals (28 female) between the ages of 17 and 34 years (*M* = 19.90, *SD* = 3.20) were recruited to participate in this study. An *a priori* power analysis was conducted using G*Power (version 3.1.9.7) to determine the required sample size. Assuming a mixed-design ANOVA with a within-between interaction, a medium effect size (*f* = 0.25), an alpha level of 0.05, and desired power of 0.90, the analysis indicated a minimum sample size of 46 participants. To account for potential data loss and to ensure adequate statistical power, a total of 50 participants were recruited.

The Rightward Bias group consisted of 10 males and 15 females between the ages of 17 and 34 years (*M* = 20.36, *SD* = 3.91). The Leftward Bias group consisted of 12 males and 13 females between the ages of 18 and 26 years (*M* = 19.44, *SD* = 2.26). Overall, of the 50 participants, 46 were recruited through the Psychology Department Undergraduate Participant Pool at the University of Manitoba and received course credit toward their Introductory Psychology course. The remaining four participants were recruited from the general student body at the University of Manitoba and received a CAD$10 Starbucks gift card in exchange for their participation. All experimental procedures conformed to the guidelines of the Declaration of Helsinki. This study was approved by the Research Ethics Board at the University of Manitoba, Fort Garry.

All participants reported to be right-hand dominant, which was verified by a modified version of the Edinburgh Handedness Inventory (Oldfield, 1971). In addition, all participants had normal or corrected-to-normal vision, and English as their native reading language. English as the native reading language was required for participation to control for any potential spatial biases, which may change based on an individual’s native reading language (right-to-left vs. left-to-right; Bowers and Heilman, 1980; Chokron et al., 1998; Smith et al., 2015).

### 2.2 Materials and procedure

MotionMonitor xGen software (Innovative Sports Training Inc., Chicago, IL, United States) was utilized to generate a two-dimensional target that was 7.00 × 3.50 cm in size (8.02 deg × 4.01 deg), gray in color and moved at a speed of 15 cm/s (17.19 deg/s). The target was displayed on a Dell U2414H 24-in. computer monitor, with a resolution of 1,080p and a refresh rate of 60 Hz. The monitor was placed 50 cm away from the participant.

To track participants’ hand movements, two wired infrared emitting diodes (IREDs) were placed on the index finger, two on the thumb, and two on the distal radius of the wrist, on their right hand. The four IREDs placed on the index finger and thumb were positioned 0.50 cm away from the fingertips. The IRED wires were made of flexible, light-weight material and were taped to the participants’ shoulders to prevent any interference throughout the study. Gaze position was recorded by an EyeLink II head-mounted eye-tracking system (250 Hz sampling rate, spatial resolution < 0.05 deg; SR research Ltd., Mississauga Ontario, Canada). Three additional IREDs were placed on the EyeLink II headset to track the movements of the head. IRED movements were captured using an Optotrak Certus 3-D motion tracking system (100 Hz sampling rate, spatial accuracy up to 0.01 mm; Northern Digital Inc., Waterloo, Ontario, Canada). The eye-tracking system was calibrated using a 9-point calibration system. Since the EyeLink II head-mounted eye-tracking system and the Optotrak Certus 3-D motion tracking system recorded at two different frequencies, data produced by these two systems were integrated into a common frame of reference using MotionMonitor for analysis.

**FIGURE 1:**
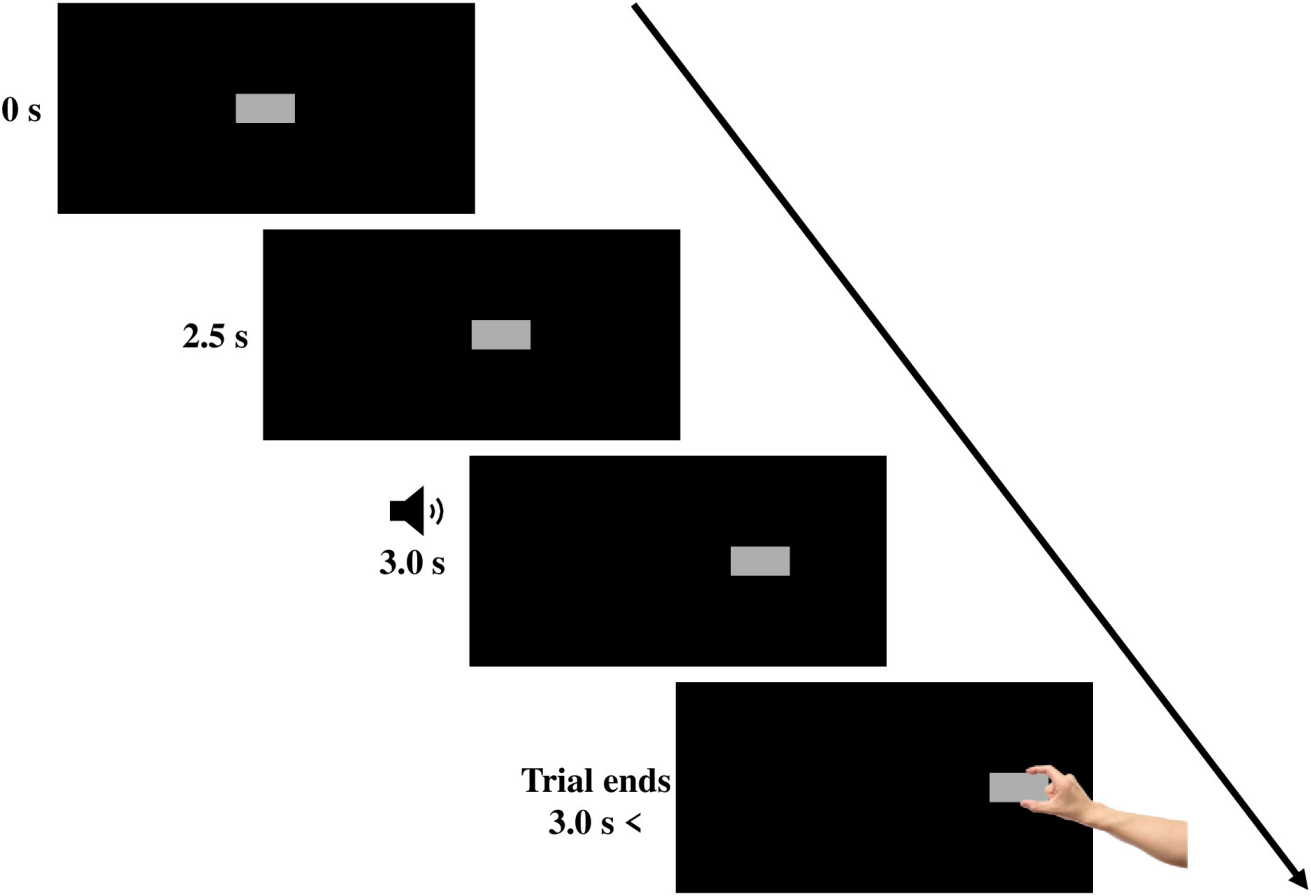
An example of a rightward-moving trial. On each trial, the target appeared in the middle of the screen. After 2.5 s, the target began translating horizontally. A tone was played after 0.5 s from target movement onset, prompting participants to begin reaching and grasping the target.

As illustrated in Figure 1, each trial began when the target block appeared. The target remained stationary in the middle of the screen for approximately 2.5 s. After 2.5 s, the target began moving horizontally. Three seconds after the beginning of the trial, a 450 Hz tone was played for a duration of 250 ms. The tone served as a “go” signal that directed the participant to reach and grasp the moving target “as quickly and naturally as possible” using their index finger and thumb. Once grasped, the target stopped in the position in which it was grasped for 2 s, and then it disappeared, indicating the end of the trial. To ensure that the target behaved in a manner like a moving three-dimensional object, the target was programed to stop its movement once the distance of one of the two IREDs placed on the index finger reached 0.50 cm from the screen. Since the IREDs were placed approximately 0.50 cm from the tip of the index finger, the target stopped at the same moment the index finger touched the screen, mimicking a real 3D moving object that stops when grasped.

Participants first completed a no-bias block of trials, in which the target translated horizontally toward the left in 50% of the trials and toward the right in the other 50% of trials, in a pseudo-randomized order. There were 24 experimental trials (12 leftward-moving and 12 rightward-moving) and 8 timing distractor trials (4 leftward-moving and 4 rightward-moving) in the no-bias block. Timing distractor trials were added to increase variability between trials to ensure that participants remained attentive throughout the entire study but were not included in the analysis. In the timing distractor trials, the timing of the “go” tone was manipulated so that it was presented at the same time the target started to move. Following the no-bias block, participants completed two “biased” blocks, where the directionality bias of the target was set to 75% in the biased direction and 25% in the opposite direction. There were 24 experimental trials in each biased block, 18 of them moved in either the right or left direction, depending on the group the participant was assigned to, while the remaining 6 trials moved in the opposite direction (3:1 ratio of biased to non-biased direction trials). In addition, there were 8 timing distractor trials in the biased group conditions. The target moved toward the direction of the bias in 6 of the timing distractor trials, and in the opposite direction in the remaining 2 trials (3:1 ratio of biased to non-biased distractor trials).

The order in which the trials were organized was pseudorandomized. In the no-bias block, no more than two trials involving target movement in the same direction occurred consecutively. In the two biased blocks, trials moving in the “non-biased” direction did not appear consecutively. The experimental trials within each block of trials were averaged to create a mean gaze deviation, per block for each participant. Gaze deviation refers to the horizontal displacement of the participant’s gaze relative to the target’s midline during the anticipatory period.

Each data collection session began with participants signing a consent form detailing information regarding the study. Participants then completed a brief questionnaire reporting age, normal vision, and handedness. Participants were seated on a height-adjustable chair with their chin placed in a chinrest, to keep their heads steady for the duration of the study. Participants were fitted with the tracking equipment and the system calibration and validation commenced. To ensure proper calibration of the system, a calibration check was conducted, where participants were required to stare at a 2 mm dot presented in the middle of the screen for a period of 8 s. Acceptable gaze-error ranges were 0.5 cm or less for the X-coordinate and 1.0 cm or less for the Y-coordinate. Similar error ranges have previously been utilized by Langridge and Marotta (2017, 2020, 2021), Bulloch et al. (2015), and Thulasiram et al. (2020). If the Eyelink calibration check indicated error rates outside of the accepted margin of error, the Eyelink was recalibrated. To ensure accuracy, Eyelink calibration checks were conducted every 16 trials (twice/block; six calibration checks over the entire data collection session). Each session lasted roughly 90 min.

### 2.3 Data setup and analyses

Oculomotor behaviors of interest in this study were categorized into one of two groups: anticipatory behavior and reactive behavior. Anticipatory behavior is defined as behavior occurring prior to the target’s movement onset, the period of the trial during which the target was present, but its eventual movement direction was not known. Whereas reactive behavior is defined as behavior that occurred after the target has begun moving and participants have perceived its movement direction. This categorization allowed us to achieve two goals: examining the influence of directionality bias on anticipatory behavior and investigating how reactive behavior differs in the case of anticipatory behavior changing.

Since we were interested in analyzing the influence of the directionality bias manipulation on participants’ oculomotor behavior, we first wanted to ensure that any initial bias participants may have had for any reason other than the experimental manipulation was measured and considered. To ensure that there were no significant differences between groups or within blocks for gaze measurement accuracy, a 2 (Directionality Bias Group: leftward or rightward) × 3 (Trial Block: unbiased block, biased block 1, and biased block 2) mixed analysis of variance (ANOVA) was conducted, with directionality bias as a between-subjects factor, and trial block as a within-subject factor on average displacement error as the dependent variable. Average displacement error is defined as the value of the observed error rate obtained at each calibration check, averaged. Moreover, a second 2 (Directionality Bias Group: leftward or rightward) × 3 (Trial Block: unbiased block, biased block 1, and biased block 2) mixed ANOVA was conducted, with directionality bias as a between-subjects factor, and trial block as a within-subject factor on average absolute error as the dependent variable. Average absolute error is defined as the absolute value of observed error rate obtained at each calibration check, averaged. All gaze measurement accuracy metrics were computed based on the horizontal eye position, as the experimental manipulation and target motion were restricted to the horizontal dimension.

To examine the effects of directionality bias on anticipatory behavior, a 2 (Directionality Bias Group: leftward or rightward) × 2 (Trial Block: biased block 1 and biased block 2) mixed ANOVA was conducted, with directionality bias direction as a between-subjects factor, and trial block group as a within-subject factor on gaze deviation as the dependent variable for anticipatory behavior. Gaze deviation is defined as the average distance between participants’ gaze direction and the target’s horizontal midline during the first 2.5 s of each trial. Gaze deviations were standardized such that positive values indicated gaze positions ahead of the target’s midline in the direction of the eventual target movement, and negative values indicated gaze positions trailing the target’s horizontal midline.

To examine the influences of changes in anticipatory behavior on reactive behavior, a 2 (Directionality Bias Group: leftward or rightward) × 2 (Trial Block: biased block 1 and biased block 2) mixed ANOVA was conducted, with directionality bias direction as a between-subjects factor, and trial block group as a within-subject factor for each of the three following reactive behavior dependent variables: first catch-up saccade after target’s movement onset, first fixation after the first catch-up saccade, and fixation at reach onset. The first catch-up saccade is identified through an automatic method combining acceleration and velocity thresholds. It was defined as the initial eye movement occurring at least 100 ms after the target’s movement onset, with a velocity surpassing 35°/s. The saccade also included adjacent frames where the acceleration surpassed 1,000°/s^2^ (Krauzlis and Miles, 1996). The 100 ms delay in saccade detection was included to ensure that a saccade was initiated after the target’s movement onset had become perceptible (Orban de Xivry and Lefèvre, 2007). The first fixation following the first catch-up saccade and the fixation immediately preceding or co-occurring with the initiation of the reaching movement were determined based on the dispersion-threshold identification (I-DT) algorithm (Salvucci and Goldberg, 2000). More specifically, fixations were defined as a pause in eye movements for at least 100 ms, with a maximum dispersion threshold of 1 cm. The first fixation following the catch-up saccade was analyzed to examine whether variations in anticipatory behavior, emerging before the onset of target motion, are reflected in the spatial characteristics of subsequent target tracking. Specifically, we investigated whether enhanced anticipatory behavior facilitates more precise tracking, as indicated by a fixation positioned closer to or ahead of the target’s midline during the reaction phase. Furthermore, we analyzed the fixation occurring immediately before or concurrently with reach onset, as previous research suggests that this fixation reflects the selection of the grasp location (Cavina-Pratesi and Hesse, 2013).

Given our interest in assessing the influence of directionality bias, we sought to ensure that any initial differences in oculomotor behavior between the Rightward and Leftward bias groups were not the result of random variation due to sampling error. To address this, we conducted an independent samples *t*-test, or, when appropriate, its non-parametric equivalent, on each dependent variable during the no-bias condition, comparing the Rightward and Leftward bias groups. The no-bias condition was not included in the aforementioned mixed ANOVAs because it served solely as a baseline assessment rather than as an experimental condition relevant to our hypotheses.

Furthermore, to examine the influence of directionality bias and potential changes in anticipatory or reactionary oculomotor behavior on movement kinematics, we conducted a paired-samples *t*-test, or, when necessary, its non-parametric equivalent, on each of the following dependent variables: maximum reaching velocity, reach duration, and reach latency. Maximum reaching velocity refers to the peak speed achieved during the reach, reach duration refers to the total time from movement onset to target grasp, and reach latency refers to the time between tone presentation and movement onset. Movement onset was defined as the point at which wrist velocity reached 5 cm/s. These analyses were restricted to within-subject comparisons across the two biased trial blocks. Since right-handed reaching toward a target moving rightward differs biomechanically from reaching toward a target moving leftward, requiring a cross-body movement, we did not compare the Rightward and Leftward Bias groups. Any between-subject differences would likely reflect these inherent biomechanical asymmetries rather than effects attributable to the manipulation in this study and thus would not support meaningful interpretation.

To determine if the amount of anticipatory gaze prior to the target’s movement predicted fixation behavior after target movement, linear mixed-effect models (LMMs) using the lme4 package in R (Bates et al., 2015) were fitted to the (i) amplitude and (ii) duration of the first catch-up saccade occurring after target movement, (iii) first fixation after the first catch-up saccade, and (iv) fixation at reach onset. In addition to anticipatory gaze, trial block (biased block 1 and biased block 2) and directionality bias (left and right) were included as controlled-for fixed effect predictors. Participant number was included as a random intercept to account for within participant variability.

Inspections of the LMM residuals revealed severe violation of the assumptions of normality, large Cook’s Distance values indicating a disproportionate influence of several data points on model estimates, and moderately high autocorrelation. To address these violations, the original LMMs were replaced with robust mixed-effects models (RMMs) using the robustlmm package in R (Koller, 2016). RMMs are specifically designed to address the observed limitations, and boast tolerance to non-normal samples, reduction of influence of disproportionately influential data points, and are less sensitive to the presence of autocorrelation. RMMs do not provide the use of traditional hypothesis testing or *p*-values, and therefore the significance of the anticipatory gaze predictor was assessed using 95% confidence intervals (CIs); the predictor was considered statistically significant if 0 was not contained in its 95% CI.

## 3 Results

### 3.1 Excluded data

Experimental data were excluded from analysis if the participant executed the task incorrectly (i.e., initiated the reach prior to the presentation of the tone, failed to grasp the target at its actual on-screen location or failed to grasp the target prior to its movement off the screen) or if data was lost due to equipment failure. A total of 6.10% of trials were excluded due to one of these two reasons.

### 3.2 Gaze measurement accuracy

The average displacement error for all participants across all blocks was −0.03 cm (*SD* = 0.24) in the horizontal axis. Across all three blocks, the average displacement error was 0.08 cm (*SD* = 0.25) for participants in the Leftward bias group, and 0.02 cm (*SD* = 0.21) for participants in the Rightward bias group. The analysis indicated no significant main effect of directionality bias group on average displacement error, *F*(1, 48) = 3.97, *p* = 0.052, η_*p*_^2^ = 0.07; no significant main effect of trial block on average displacement error, *F*(1.61, 77.29) = 1.00, *p* = 0.356, η_*p*_^2^ = 0.02; and no significant interaction between directionality bias group and trial block, *F*(1.61, 77.29) = 1.00, *p* = 0.355, η_*p*_^2^ = 0.02.

The average absolute error combined among all participants, and across all blocks, was 0.32 cm (*SD* = 0.12) in the horizontal axis. Across all three blocks, the average absolute error was 0.33 cm (*SD* = 0.12) for participants in the Leftward bias group, and 0.31 cm (*SD* = 0.12) for participants in the Rightward bias group. Results revealed no significant main effect of directionality bias group on absolute error rate, *F*(1, 48) = 0.30, *p* = 0.582, η_*p*_^2^ = 0.01; no significant main effect of trial block on absolute error rate, *F*(2, 96) = 2.21, *p* = 0.115, η_*p*_^2^ = 0.04; and no significant interaction between directionality bias group and trial block, *F*(2, 96) = 0.49, *p* = 0.615, η_*p*_^2^ = 0.01. Together, these results confirm the average displacement error and average absolute gaze error did not differ between the two experimental groups and did not change between the three blocks of experimental trials.

### 3.3 Potential initial unmanipulated biases

During the no-bias block of trials, we observed similar average anticipatory gaze behavior between the two groups; the Rightward bias group had mean gaze of 0.08 cm ahead of the target’s horizontal midline (*SD* = 0.24) and the Leftward bias group had mean gaze of 0.04 cm ahead of the target’s horizontal midline (*SD* = 0.24). Results of the independent *t*-test indicated no significant difference in anticipatory gaze behavior between the two groups, *t*(48) = −0.58, *p* = 0.562. This shows that during the non-biased condition, participants’ anticipatory gaze was directed toward the center of the target prior to its movement onset in both groups (approximately 0.06 cm away from the target’s center). These results confirm the lack of inherent differences between the Leftward and Rightward bias groups, prior to their exposure to the biased conditions.

### 3.4 Anticipatory gaze behavior

**FIGURE 2:**
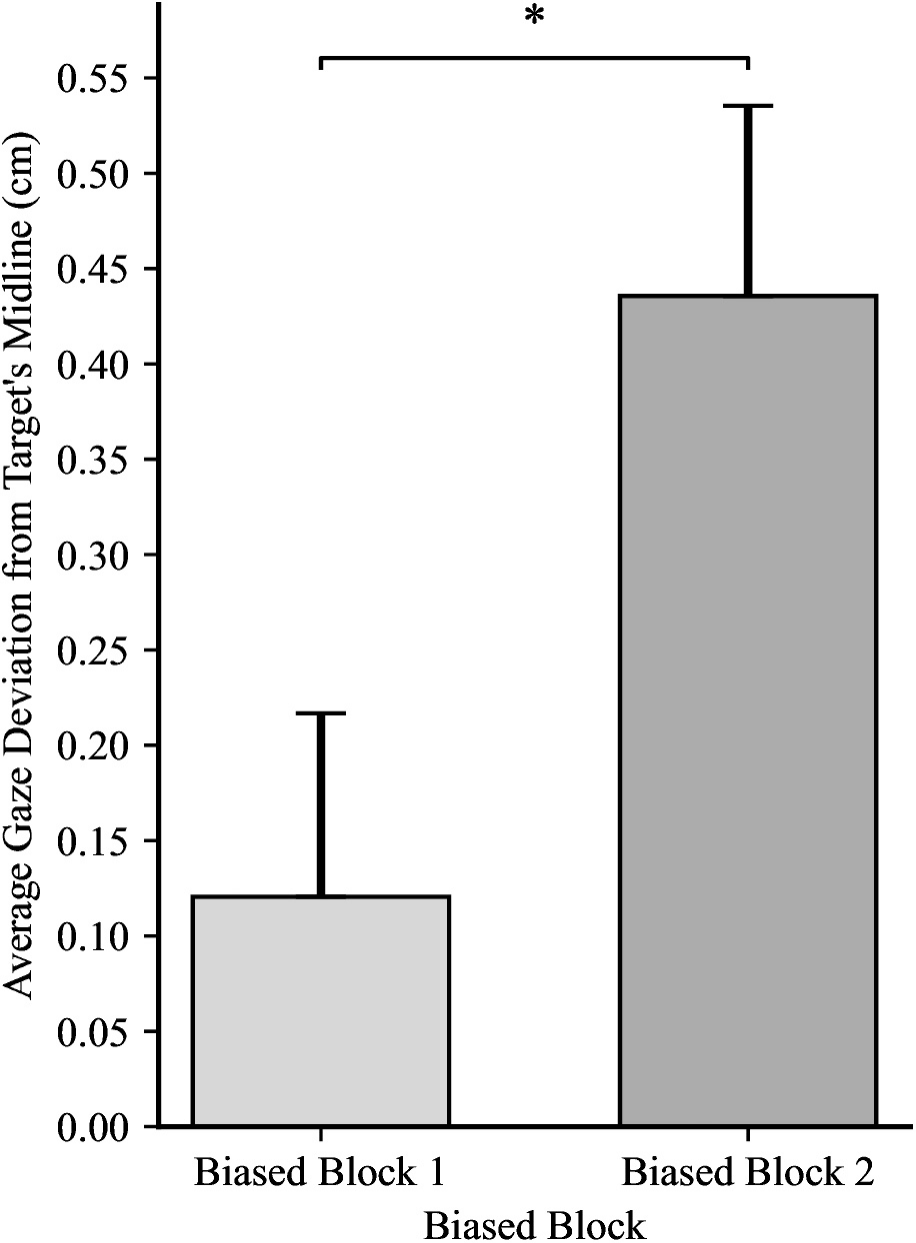
Average gaze deviation in biased block 1 and biased block 2, with the data for the between-subjects groups (directionality bias: leftward and rightward) collapsed. Positive values indicate deviation in the direction of future target movement. Error bars represent standard error of the mean. *p < 0.01.

Confident in the lack of any inherent directional bias in participants’ gaze unrelated to our experimental manipulation, we examined the effects of directionality bias on anticipatory gaze behavior. The main effect of Directionality Bias Group was not significant, *F*(1, 48) = 3.43, *p* = 0.070, η_*p*_^2^ = 0.07, indicating that anticipatory gaze behavior was not affected by the direction of the bias. However, there was a significant main effect of Trial Block, *F*(1, 48) = 15.84, *p* < 0.001, η_*p*_^2^ = 0.25. Collapsing across the two directionality-biased-blocks, participants in Biased Block 1 displayed a mean gaze deviation of 0.12 cm ahead of the target’s horizontal midline (*SD* = 0.68). Whereas participants in biased block 2 displayed a mean gaze deviation of 0.44 cm behind the target’s horizontal midline (*SD* = 0.71). This indicates that participants adapted their gaze strategy as the experiment progressed, with gaze during later trials being positioned more distant from the center of the target than earlier trials, as illustrated in Figure 2. The interaction between directionality bias direction and trial block was not significant, *F*(1, 48) = 0.00, *p* = 0.890, η_*p*_^2^ = 0.00, suggesting that the change in anticipatory gaze behavior observed as participants completed more biased trials was not differentially impacted by the bias direction.

### 3.5 First catch-up saccade

**FIGURE 3:**
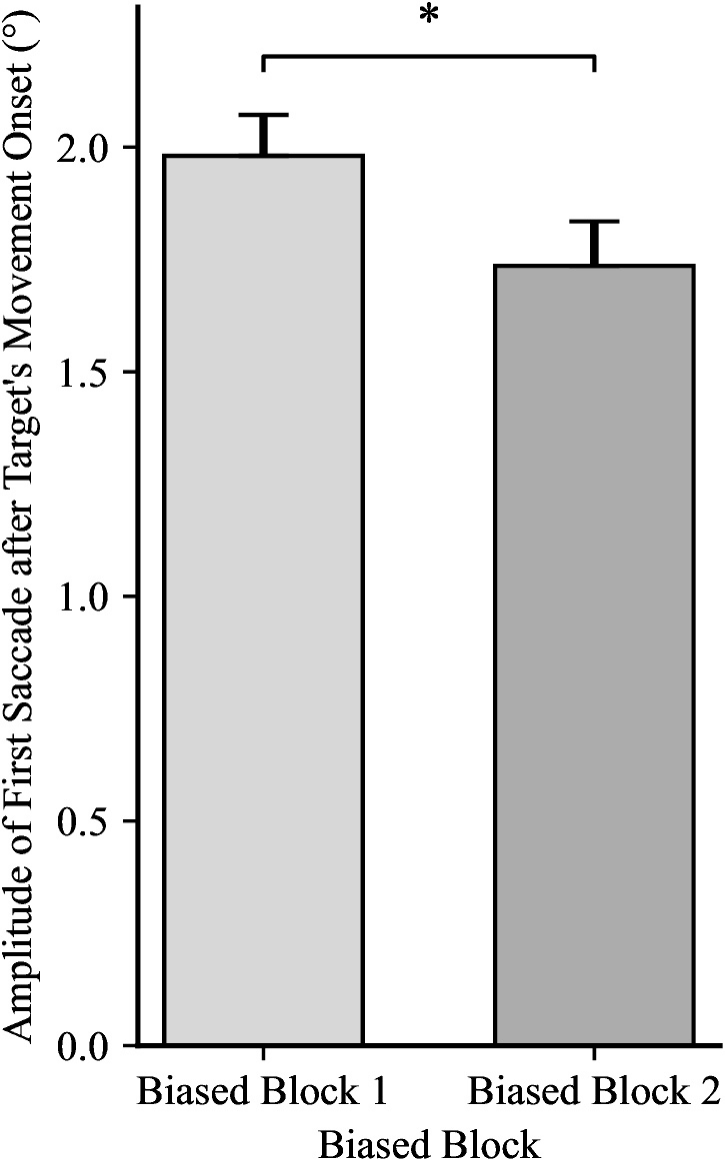
Catch-up saccade amplitudes during both Trial Blocks, with the data for the between-subjects groups (directionality bias: leftward and rightward) collapsed. Error bars represent standard error of the mean. *p < 0.05.

For the amplitude of the first catch-up saccade after the target’s movement onset, a 2 × 2 mixed ANOVA yielded a significant effect for Trial Block, *F*(1, 48) = 7.39, *p* = 0.009, η_*p*_^2^ = 0.13, showing that the amplitude of the catch-up saccade shrunk from biased block 1 to biased block 2, as illustrated in Figure 3. However, the ANOVA yielded no significance for Directionality Bias Group, *F*(1, 48) = 0.52, *p* = 0.476, η_*p*_^2^ = 0.01, and no significant interaction, *F*(1, 48) = 0.01, *p* = 0.908, η_*p*_^2^ = 0.00. An independent samples *t*-test yielded no significant differences between the two Directionality Bias Groups during the no-bias block, *t*(48) = −0.43, *p* = 0.668.

Additionally, for the duration of the first catch-up saccade after the target’s movement onset, a 2 × 2 mixed ANOVA yielded significance for Trial Block, *F*(1, 48) = 5.77, *p* = 0.020, η_*p*_^2^ = 0.11, revealing shorter saccade duration in biased block 2 (*M* = 28.20 ms, *SD* = 6.23) than in biased block 1 (*M* = 30.11 ms, *SD* = 5.83). However, there was no significant difference for Directionality Bias Group, *F*(1, 48) = 1.15, *p* = 0.288, η_*p*_^2^ = 0.02, and no significant interaction, *F*(1, 48) = 1.65, *p* = 0.205, η_*p*_^2^ = 0.03. An independent samples *t*-test yielded no significant differences between the two Directionality Bias Groups during the no-bias block, *t*(48) = 0.70, *p* = 0.485.

### 3.6 Fixation after catch-up saccade

**FIGURE 4:**
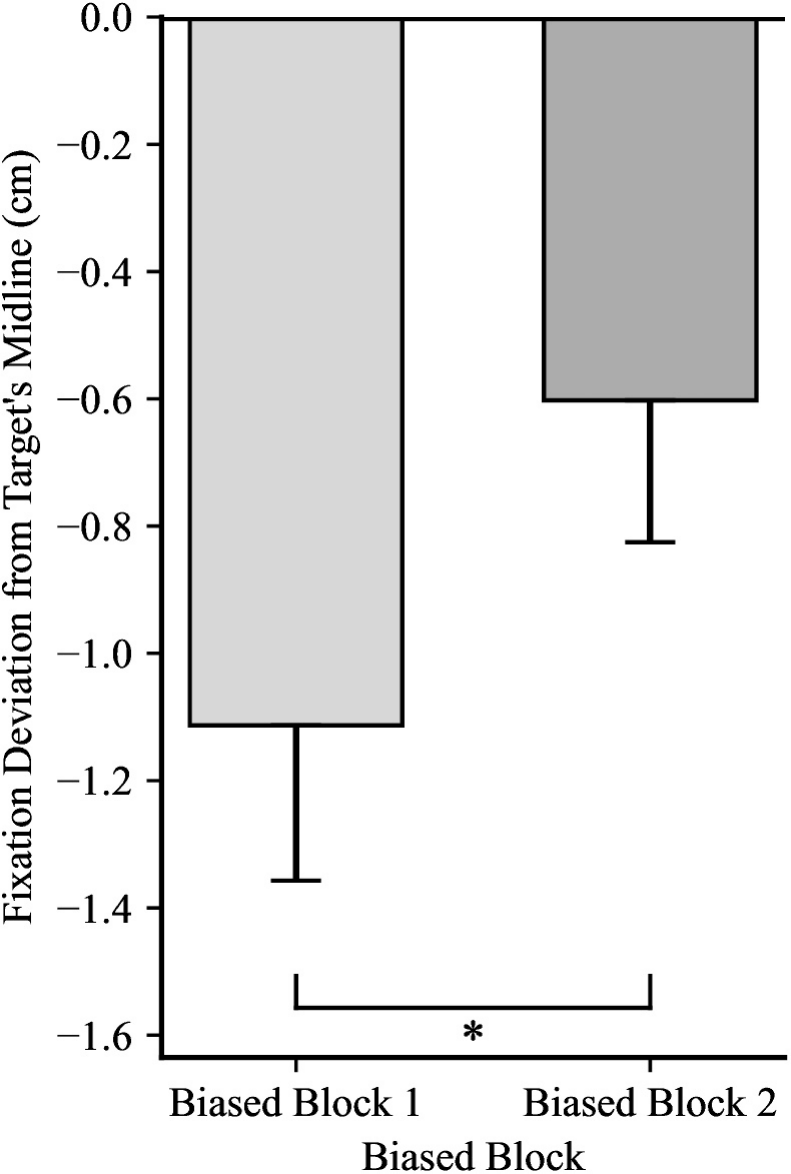
Fixation deviation between the Leftward Bias and Rightward Bias groups, while collapsing across Trial Block. Y-axis represents the deviation of the first fixation following the catch-up saccade from the target’s midline, with negative values indicating fixation trailing the target’s midline and positive values indicating fixation ahead of it. Error bars represent standard error of the mean. *p < 0.001.

**FIGURE 5:**
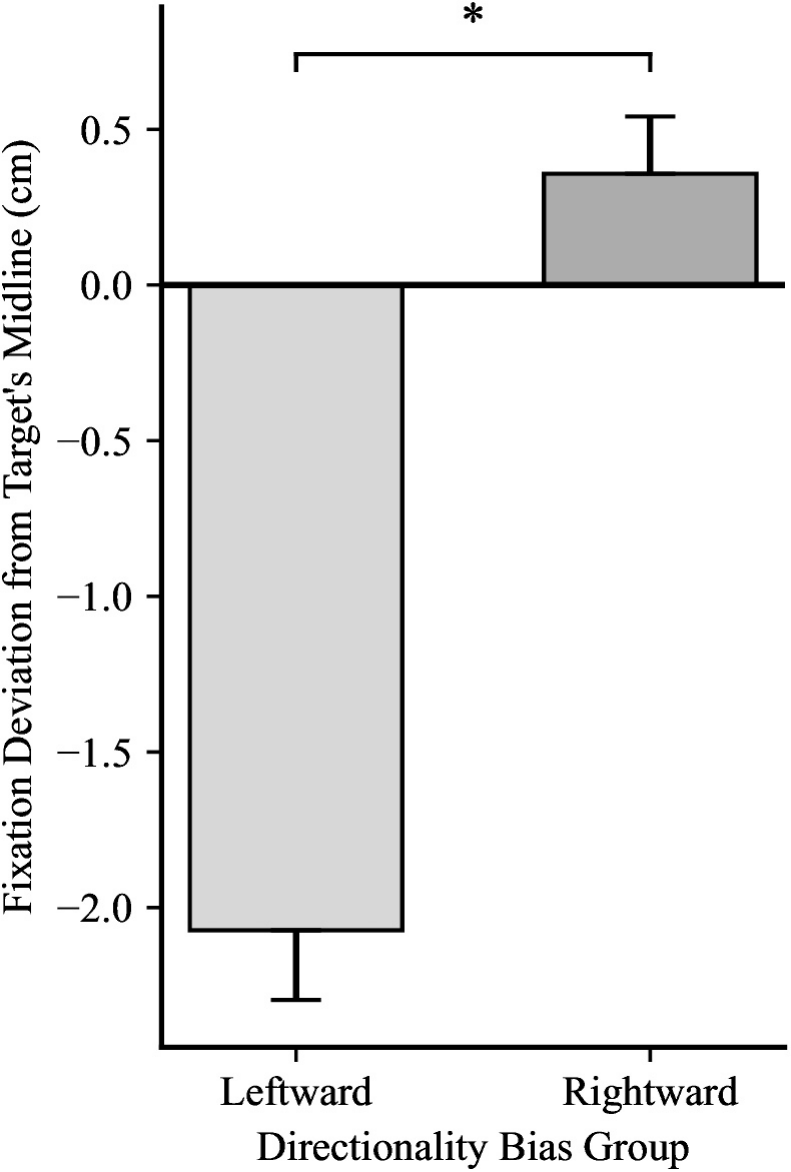
Fixation deviation across the two Trial Blocks, collapsing across Directionality Bias group. Y-axis represents the deviation of the first fixation following the catch-up saccade from the target’s midline, with negative values indicating fixation trailing the target’s midline and positive values indicating fixation ahead of it. Error bars represent standard error of the mean. *p < 0.001.

For the first fixation after the catch-up saccade, a 2 × 2 mixed ANOVA yielded significance for Directionality Bias Group, *F*(1, 48) = 70.35, *p* < 0.001, η_*p*_^2^ = 0.59, and for Trial Block, *F*(1, 48) = 16.85, *p* < 0.001, η_*p*_^2^ = 0.26, but no significant interaction, *F*(1, 48) = 0.66, *p* = 0.199, η_*p*_^2^ = 0.03, as illustrated in Figures 4, 5, respectively. In biased block 1, the fixation position was ahead of the target’s midline by 0.18 cm (*SD* = 0.94) in the Rightward Bias Group but trailed the target’s midline by 2.41 cm (*SD* = 1.30) in the Leftward Bias Group. In biased block 2, the fixation position was ahead of the target’s midline by 0.53 cm (*SD* = 1.01) in the Rightward Bias Group but trailed the target’s midline by 1.74 cm (*SD* = 1.13) in the Leftward Bias Group. Collapsing across the Directionality Bias Groups, the distance between the fixation position and target’s midline shrunk from biased block 1 to biased block 2. This shrinkage pattern persisted for each Directionality Bias Groups, with the Rightward Bias Group showing better target tracking than the Leftward Bias Group. An independent samples *t*-test yielded no significant differences between the two Directionality Bias Groups during the no-bias block, *t*(48) = −0.22, *p* = 0.829.

### 3.7 Fixation at reach onset

**FIGURE 6:**
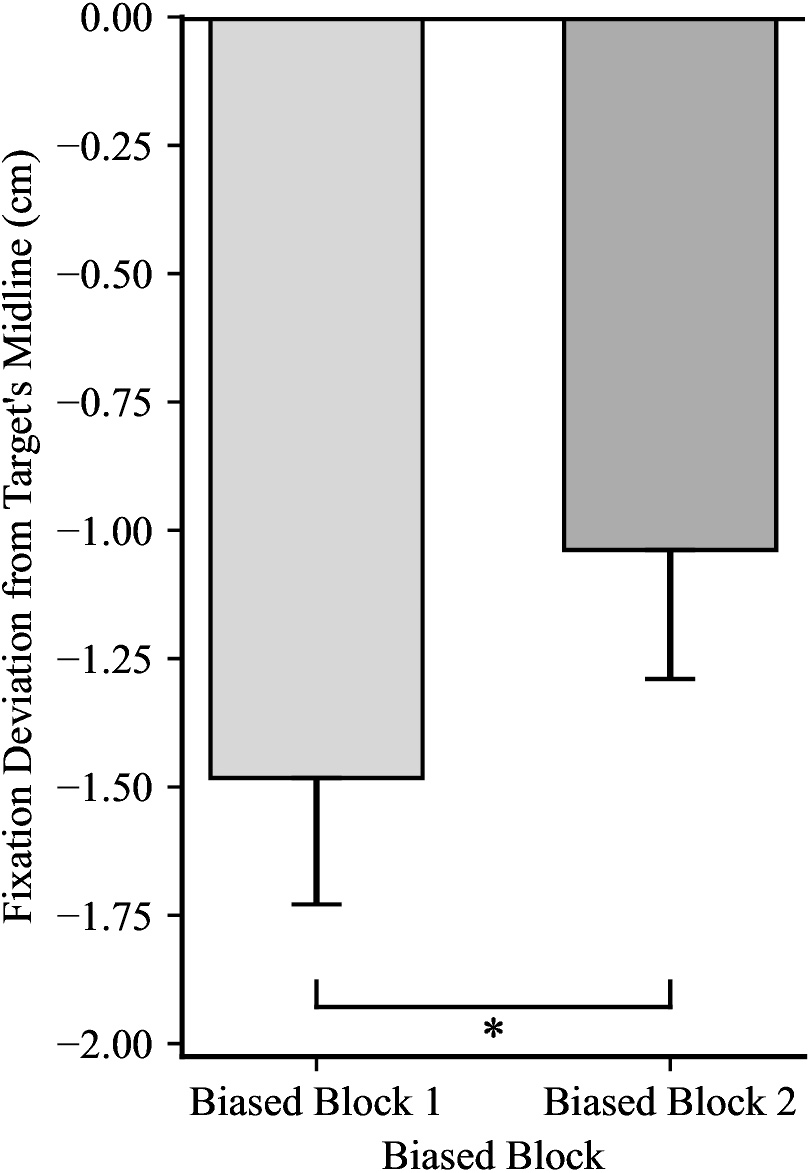
Effect of trial block on fixation deviation at the time of reach onset, collapsing across Directionality Bias group. Y-axis represents the deviation of the fixation from the target’s midline, with negative values indicating fixation trailing the midline. Error bars represent standard error of the mean. *p < 0.01.

For the fixation at reach onset, a 2 × 2 mixed ANOVA yielded significance for Trial Block, *F*(1, 48) = 9.18, *p* = 0.004, η_*p*_^2^ = 0.16, showing that despite fixations falling behind the target’s midline in both trial blocks, fixations were closer to the target’s midline in biased block 2 compared to biased block 1, as illustrated in Figure 6. However, there was no significant effect for Directionality Bias Group, *F*(1, 48) = 0.75, *p* = 0.391, η_*p*_^2^ = 0.02, and no significant interaction, *F*(1, 48) = 2.24, *p* = 0.141, η_*p*_^2^ = 0.05. Moreover, an independent samples *t*-test yielded no significant differences between the two Directionality Bias Groups during the no-bias block, *t*(48) = −0.50, *p* = 0.621.

### 3.8 Kinematic variables

For maximum reaching velocity, a Wilcoxon signed-rank test indicated a significant difference between trial blocks, *z* = −2.36, *p* = 0.018, with participants exhibiting lower peak reaching velocity in biased block 2, with an average of 83.31 cm/s (*SD* = 31.77) compared to biased block 1, with an average of 86.54 cm/s (*SD* = 33.26).

For reach duration, a Wilcoxon signed-rank test showed no significant difference between biased block 1 (*M* = 0.54, *SD* = 0.09) and biased block 2 (*M* = 0.54, *SD* = 0.09), *z* = −0.56, *p* = 0.576.

For reach latency, a paired-samples *t*-test yielded no significant difference between biased block 1 (*M* = 0.97, *SD* = 0.07) and biased block 2 (*M* = 0.96, *SD* = 0.07), *t*(49) = 1.20, *p* = 0.235.

### 3.9 Robust mixed-effects models

The fixed-effect estimates are provided in Tables 1A–D.

**TABLE 1:** Fixed-effect estimates for (A) first catch-up saccade amplitude, (B) first catch-up saccade duration, (C) first fixation after catch-up saccade, and (D) fixation at reach onset deviation.

Fixed effect	Estimate	Std. error	Lower 95% CI	Higher 95% CI
**(A) First catch-up saccade amplitude**				
Intercept	1.83	0.13	1.58	2.09
Anticipatory gaze	−0.20	0.10	−0.39	−0.01
Biased block	−0.18	0.08	−0.34	−0.02
Directionality bias	0.17	0.17	−0.17	0.51
**(B) First catch-up saccade duration**				
Intercept	28.88	1.24	26.44	31.32
Anticipatory gaze	−1.02	1.00	−2.98	0.93
Biased block	−1.49	0.89	−3.24	0.27
Directionality bias	1.81	1.63	−1.38	5.00
**(C) First fixation after catch-up saccade**				
Intercept	0.17	0.20	−0.23	0.56
Anticipatory gaze	0.27	0.13	0.01	0.53
Biased block	0.37	0.10	0.18	0.57
Directionality bias	−2.47	0.27	−3.00	−1.93
**(D) Fixation at reach onset deviation**				
Intercept	−1.60	0.33	−2.25	−0.94
Anticipatory gaze	0.12	0.14	−0.16	0.40
Biased block	0.25	0.10	0.06	0.44
Directionality bias	0.43	0.47	−0.48	1.35

Estimates represent unstandardized coefficients from robust linear mixed models with Participant included as a random intercept. Predictors whose 95% confidence intervals do not contain 0 are interpreted as statistically significant. For the categorical predictors Biased Block and Directionality Bias, the first of the two biased blocks and the rightward directionality bias served as reference levels, respectively.

#### 3.9.1 First catch-up saccade amplitude

95% CIs for the anticipatory gaze predictor did not include 0, and therefore anticipatory gaze was determined to be a significantly negative predictor of first catch-up saccade amplitude. A larger amount anticipatory gaze prior to the movement onset of the target significantly predicted smaller catch-up saccade amplitude (Table 1A).

#### 3.9.2 First catch-up saccade duration

95% CIs for the anticipatory gaze predictor contained 0, and therefore anticipatory gaze was determined non-significant (Table 1B).

#### 3.9.3 First fixation after the first catch-up saccade

95% CIs for the anticipatory gaze predictor did not include 0, and therefore anticipatory gaze was determined to be a significantly positive predictor of the deviation from first fixation after catch-up saccade to target midline A larger amount anticipatory gaze prior to the movement onset of the target significantly predicted a larger deviation between the fixation location and target midline (Table 1C). In other words, the more a participant engaged in anticipatory behavior prior to target movement, the more their first fixation after the catch-up saccade was ahead of the target’s midline, suggesting better tracking performance.

#### 3.9.4 Fixation at reach onset

95% CIs for the anticipatory gaze predictor contained 0, and therefore anticipatory gaze was determined non-significant (Table 1D).

## 4 Discussion

The present study aimed to investigate the effects of directionality bias on anticipatory and reactive gaze behavior during a reaching-and-grasping task where the only indication regarding direction of future target movement was movement history. Participants were presented with a target with a manipulated directionality bias, causing it to move predominantly either leftward or rightward, depending on the experimental condition. Our findings indicate that participants’ anticipatory gaze behavior is sensitive to the directionality bias of a moving target, supporting our hypothesis that under uncertain conditions, the visuomotor system can utilize movement history to predict future target positions during the execution of goal-directed behavior. We observed a significant increase in anticipatory gaze deviation in the second block of biased trials compared to the first block, indicating that participants effectively adapted to the directionality bias of the target over time. Unlike previous studies that focused on trajectory identification in action tasks (Bulloch et al., 2015; Diaz et al., 2013; Hayhoe et al., 2012; Thulasiram et al., 2020) or directionality bias utilization in perceptual tasks (Damasse et al., 2018; Montagnini et al., 2010; Notaro et al., 2019; Santos and Kowler, 2017), our research underscores the visuomotor system’s capacity to mitigate uncertainty by leveraging learned statistical patterns to successfully execute goal-directed actions when the eventual direction of target movement is unknown.

Furthermore, in this study, we examined the influence of anticipatory behavior on subsequent tracking behavior. Results revealed a reduction in the amplitude of catch-up saccades during biased block 2 relative to biased block 1. Notwithstanding this decrease in saccade amplitude, both the fixation immediately following the catch-up saccade and the fixation at reach onset were positioned closer to, or ahead of, the target’s midline in biased block 2 compared to biased block 1, reflecting improved tracking performance.

To directly assess the role of anticipatory behavior in shaping reactive oculomotor responses, we ran robust mixed-effects analyses, which revealed that anticipatory gaze significantly predicted both the amplitude of the first catch-up saccade and the spatial position of the first fixation following that saccade, providing converging evidence that early predictive behavior facilitated later oculomotor control (Simó et al., 2005). The reduction in the distance between fixation position following the first catch-up saccade and the target’s midline across Biased Blocks is consistent with increasingly accurate anticipatory gaze placement prior to target movement onset. When gaze is positioned closer to the expected future location of the target, less corrective adjustment is required once the target begins moving, resulting in both smaller amplitude of the catch-up saccades and fixation locations that are more closely aligned with the target’s trajectory.

This interpretation aligns with prior work demonstrating that anticipatory eye movements reduce subsequent position error and improve tracking efficiency by minimizing the need for reactive correction following motion onset (Kowler, 1989; Barnes and Asselman, 1991; Diaz et al., 2013). Taken together, the reduction fixation deviation from target’s midline following the first catch-up saccade and the correlation between anticipatory gaze and fixation position following the catch-up saccade suggest that improvements in reactive tracking were directly shaped by where gaze was positioned prior to target motion. As participants increasingly directed their gaze toward the anticipated future path of the target, reactionary oculomotor tracking behavior improved, requiring less online corrective adjustments. Finally, although anticipatory gaze did not significantly predict fixation at reach onset, fixation placement at reach onset nevertheless improved across biased blocks, suggesting that anticipatory strategies informed subsequent grasp planning even if not in a direct linear manner.

In addition to changes in oculomotor behavior, we observed changes in the kinematic profile of the reaching movement as participants completed more biased trials. More specifically, maximum reaching velocity significantly decreased from biased block 1 to biased block 2, with no significant differences observed for reach latency or reach duration. A reduction in peak velocity, in the absence of changes in movement onset timing or overall movement duration, is commonly interpreted as reflecting a reduced reliance on rapid online corrective adjustments during movement execution rather than a slowing of action planning or a more cautious movement strategy (Elliott et al., 2001; Saunders and Knill, 2004).

In the context of the present task, this pattern is consistent with the interpretation that improved anticipatory gaze placement reduced spatial uncertainty once the target began moving, allowing the reach to be guided more smoothly. Notably, the absence of significant changes in reach latency suggests that anticipatory gaze did not simply facilitate earlier movement initiation, but instead influenced how the movement was controlled following onset.

This dissociation aligns with previous work demonstrating that predictive visual information primarily affects movement execution and online control, rather than movement initiation (Saunders and Knill, 2004), supporting the view that learned directionality bias contributes to more efficient goal-directed reaching by reducing reliance on online corrective control. The changes in the kinematic profile of the reaching movement also support the claim made by Bulloch et al. (2015) positing that anticipating the direction of movement of the target by looking ahead in its eventual path eliminates the “cognitive effort” that must be exerted to catch up to and continue following a target after its movement onset.

This study demonstrates the functional relationship between learned environmental statistics and visuomotor control. Despite the lack of any cues about a target’s eventual movement direction, except its movement history, participants exhibited progressively stronger anticipatory behavior across biased blocks, with this anticipatory behavior being a significant predictor of aspects of subsequent tracking behavior. This suggests that the visuomotor system made active use of implicitly acquired probabilistic knowledge to facilitate goal-directed action. This aligns with the idea that dorsal-stream control of action can draw upon representations encoded within ventral memory systems when circumstances demand predictive processing (Goodale, 2011; Milner and Goodale, 2006; Monaco et al., 2019). Our findings therefore extend previous research by demonstrating that predictive integration of movement history is expressed not merely in perceptual decisions (Betti et al., 2022; Notaro et al., 2019), but also in visuomotor contexts, thereby increasing one’s chances of successfully completing the goal-directed behavior.

The observed pattern of reactive gaze behavior further supports the role of prediction in guiding target tracking. Our robust mixed-effects analyses revealed that anticipatory gaze significantly predicted both the amplitude of the first catch-up saccade and the spatial position of the subsequent fixation. In other words, when participants exhibited stronger anticipatory behavior prior to target movement onset, they required smaller corrective saccades and achieved more optimal fixation placement once the target began moving. This pattern aligns with claims made by our lab, Bulloch et al. (2015) and Thulasiram et al. (2020), suggesting that looking ahead reduces the “effort” required to catch up to a moving stimulus.

Critically, our findings demonstrate that this predictive efficiency emerged in the absence of any explicit cues; participants inferred the likely direction of the target based solely on its movement history. This reflects implicit learning, in which environmental regularities are extracted and used to guide behavior (Fiser and Aslin, 2002; Turk-Browne, 2012). Importantly, related work indicates that environmental statistics can be learned and expressed through multiple behavioral channels without necessitating a direct correspondence between them. For instance, Pasturel et al. (2020) demonstrated that observers form internal representations of probabilistic motion patterns that influence both anticipatory eye movements and explicit judgments about target direction. However, these two behavioral expressions were not strongly correlated across individuals. This dissociation suggests that learned statistical patterns can be utilized flexibly to guide behavior in a task-dependent manner, without requiring that such information be uniformly accessible or expressed across behavioral domains. Within this framework, the present findings extend prior work by demonstrating that implicitly acquired movement statistics are sufficient to support predictive visuomotor behavior, which in turn shapes reactive behavior during the execution of goal-directed actions.

The distinction between early tracking behaviors, such as the first catch-up saccade after target movement onset and the fixation that follows it, and later tracking behavior, such as the fixation at reach onset, further clarifies how prediction shapes visuomotor behavior. Although anticipatory gaze significantly predicted early tracking behaviors, its influence on fixation at reach onset was less direct. Since the fixation at onset of the reaching movement reflects the selection of a grasp location (Cavina-Pratesi and Hesse, 2013), this suggests that learned environmental statistics is more straightforwardly applied during the initial phase of target pursuit, whereas grasp-related gaze control incorporates both learned expectations and online visual feedback. In other words, prediction appears to play a clearer role in guiding tracking immediately after movement onset, while the fixation associated with grasping reflects a more integrated process, combining what has been learned with what is currently being seen.

The observed capacity to anticipate target’s movements without explicit cues aligns with the notion that the brain employs probabilistic models to manage sensory and motor uncertainty, with the model centering around minimizing error and maximizing reward (Cox, 1946; Körding and Wolpert, 2004, 2006; Turk-Browne, 2012). For instance, studies have shown that during sensorimotor learning, the central nervous system employs Bayesian strategies to integrate sensory information with prior distributions of task variables, thus improving motor control under uncertain conditions (Körding and Wolpert, 2004). Another study demonstrated that the motor response to uncertain visual stimuli is influenced by the brain’s probabilistic estimation of sensory states, further highlighting the reliance on Bayesian inference in visuomotor control (Izawa and Shadmehr, 2008). What is more impressive than the brain’s ability to adopt complex probabilistic mechanisms to minimize error and maximize reward is its ability to do so implicitly, without one’s awareness and perhaps prior to the manifestation of behavior indicating that statistical learning has occurred (Miyashita, 1993; Izawa and Shadmehr, 2008; Turk-Browne, 2012). Therefore, it is important to discuss the possible neural mechanisms that give humans this ability to implicitly engage in statistical learning and utilize that knowledge to guide behavior in an efficient manner. While this study did not specifically explore the neural correlates of statistical learning and anticipation in uncertain visuomotor contexts, our findings offer substantial insights into the neural mechanisms underpinning the observed behaviors.

From the perspective of the two-visual-stream hypothesis, the ability to produce anticipatory gaze behavior in uncertain visuomotor contexts can be understood as a result of the interactions between the visually guided action (dorsal) and perceptual (ventral) streams (Goodale and Milner, 1992; Milner and Goodale, 2006). Although traditionally characterized as processing distinct forms of information, substantial evidence indicates robust interactions between the streams (Budisavljevic et al., 2018) during goal-directed behavior, whether acting on three-dimensional objects (Janssen et al., 2018; Van Dromme et al., 2016) or on two-dimensional (Freud et al., 2018). Such interactions likely support the utilization of a target’s movement history to anticipate its future movement direction, with ventral-stream processing contributing contextual and historical knowledge of learned directional bias that can enhance dorsal-stream control of visually guided actions (Freud et al., 2018; Donato et al., 2020).

At the same time, emerging evidence challenges the long-standing assumption that the dorsal stream operates exclusively in a feedforward, memory-independent manner, with behavioral priming (Jax and Rosenbaum, 2007, 2009), processing of generalizable physical properties outside of motor planning contexts (Buckingham et al., 2018; Schwettmann et al., 2019), and even contributions to recognition (Goldstein-Marcusohn et al., 2024) indicating the dorsal stream’s capacity to retain and apply information from prior experiences. This evolving view is further supported by evidence that cerebellar regions maintain internal sensory-motor models that enable prediction of the consequences of one’s own actions, contributing critically to timing, coordination, and anticipatory scaling during grasping (Nowak et al., 2021). Such predictive mechanisms align with the neural architecture proposed by Kravitz et al. (2011), in which a parieto-prefrontal pathway links dorsal-stream visuomotor control to regions implicated in top-down oculomotor control (Hung et al., 2011) and spatial working memory (Goldman-Rakic, 1990). Altogether, these findings provide a plausible neural account for the anticipatory gaze behavior observed in our study, suggesting that learned statistical information derived from movement history can be applied implicitly to guide goal-directed behavior, whether through crosstalk between the ventral and dorsal streams or through intrinsic dorsal-cerebellar mechanisms capable of incorporating prior experience.

Although reaching to grasp a three-dimensional object more closely reflects everyday action, the use of two-dimensional targets, such as the target employed in this study, offers strong methodological advantages. Work from our lab, as well as other research groups, have used 2D targets to explore visual perception and action. Using two-dimensional simulations allows for more rigorous manipulation of various characteristics of the target and its environment, such as shape, size, speed, and direction of movement, enabling researchers to draw more precise and systematic conclusions. While important to recognize the differences between reaching to grasp a 3D object and reaching to grasp a 2D target, recent research studies show that certain behavioral features such as gaze behavior are similar when reaching-to-grasp a 2D object and a 3D object (Bulloch et al., 2015; Langridge and Marotta, 2017, 2021; Thulasiram et al., 2020). As such, given that our primary aim was to examine how directionality bias influences goal-directed visuomotor behavior rather than to reproduce the full mechanical demands of grasping real objects, the use of 2D targets provides a valid and effective means of isolating and characterizing the anticipatory and tracking behaviors of interest.

The present design allowed for precise control over target motion and directionality bias; however, a few limitations should be acknowledged. First, the use of a two-dimensional target, while advantageous for isolating the effects of movement history on anticipatory and reactive behavior, necessarily simplifies the perceptual and mechanical demands associated with naturalistic reaching and grasping. As a result, the extent to which the observed anticipatory gaze strategies and associated kinematic adaptations generalize to interactions with fully three-dimensional objects remains an open question. In addition, although the sample size was sufficient to detect moderate effects, the present study may have been underpowered to detect smaller effects, which may partly account for some non-significant findings and warrant caution when interpreting null results. Finally, the task involved highly constrained and predictable target trajectories, which may have facilitated the detection of implicit statistical regularities. In more complex environments, where target motion could be more variable, the ability to exploit movement history may be reduced or expressed differently. Therefore, the extent to which these findings generalize to less constrained task environments remain to be determined.

Although the present study was conducted in a controlled laboratory setting, the findings have broader implications for understanding how predictive visuomotor strategies support performance in dynamic, uncertain environments. The ability to exploit movement history to guide anticipatory gaze and reduce reliance on online corrective control is central to many real-world tasks, such as sports, tool use, and other activities that require the interception of moving objects. In such contexts, efficient gaze allocation can facilitate smoother movement execution and reduce the computational demands associated with rapid corrective adjustments. More broadly, these findings may inform the design of human-machine interfaces and virtual training environments, including those used for supervising or interacting with autonomous systems such as drones, by highlighting the importance of predictable motion statistics for supporting anticipatory control. While the current study does not directly assess performance outcomes in applied settings, it provides a behavioral foundation for understanding how learned environmental statistics can be integrated into goal-directed action to improve efficiency under uncertainty.

Future research is needed to characterize the parameters under which directionality bias can be exploited to guide goal-directed behavior. Although the present study demonstrates that learned statistical regularities of a target’s movement influence both anticipatory gaze and subsequent tracking, future work should explore the limits of this capacity. For example, manipulating factors such as the bias ratio, the degree of trial-to-trial uncertainty, or the level of task complexity could clarify the conditions under which movement history can be utilized and integrated into action planning. In addition, future work could examine the temporal evolution of anticipatory gaze within the initial stationary period, prior to the target’s movement onset, which may provide further insight into how anticipatory oculomotor behavior emerges and stabilizes prior to target movement onset. Moreover, given that anticipatory gaze reflects the allocation of visual attention (Bastiaansen and Brunia, 2001), an important extension of this study would be to examine how the use of directionality bias differs when visual information is available in foveal vs. peripheral vision. These manipulations would clarify whether the advantages of anticipatory gaze behavior demonstrated in this study generalize beyond foveal tracking and reflect a broader mechanism for integrating learned statistical information into goal-directed action. Finally, it will be valuable to determine whether the effects demonstrated in this study generalize to conditions in which detecting and applying statistical regularities is even more consequential, including curved target trajectories, temporary occlusions, delayed movement onset, or risk-reward scenarios in which accurate interception is necessary.

## 5 Conclusion

While considerable research has focused on the visuomotor system’s ability to produce anticipatory behavior in uncertain perceptual contexts, our study aimed to explore this capacity within the context of goal-directed actions. We tasked participants with reaching toward and grasping a moving target that exhibited a manipulated directionality bias. This design allowed us to examine their ability to engage in statistical learning to exploit the bias and produce anticipatory behavior in response, relying solely on the target’s movement history and without any additional cues. Our results show that the visuomotor system can implicitly exploit the directionality bias of a target and produce anticipatory gaze behavior when reaching to grasp that target. Furthermore, the results showed that anticipatory behavior did not occur in isolation, instead, modeling revealed that anticipatory behavior led to meaningful downstream consequences. Stronger anticipatory gaze predicted more efficient reactive tracking, characterized by reduced corrective saccade amplitude and improved fixation placement while tracking the target. Anticipatory gaze was also accompanied by changes in movement kinematics, suggesting that implicit statistical learning not only guides early visual anticipation but also facilitates the efficiency of the resulting reaching movement. Together, these patterns indicate that learned statistical or directional bias is functionally integrated into visuomotor control, shaping both eye movement planning and reaching movement. Future studies should also focus on determining the conditions under which a directionality bias can be utilized, as well as the minimum threshold of bias required for utilization. Notably, it is important to ascertain the extent to which the bias ratio can deviate from one before it becomes unusable.

## Data Availability

The datasets presented in this study can be found in online repositories. The names of the repository/repositories and accession number(s) can be found at: https://doi.org/10.5683/SP3/TITXEK.
